# Bladder augmentation in children: current problems and experimental strategies for reconstruction

**DOI:** 10.1007/s10354-018-0645-z

**Published:** 2018-08-06

**Authors:** Sophie Langer, Christine Radtke, Eva Györi, Alexander Springer, Martin L. Metzelder

**Affiliations:** 1 General Hospital Vienna, Clinical Department of Paediatric Surgery, Medical University of Vienna, Waehringer Guertel 18–20, 1090 Vienna, Austria; 2 General Hospital Vienna, Clinical Department of Plastic and Reconstructive Surgery, Medical University of Vienna, Waehringer Guertel 18–20, 1090 Vienna, Austria

**Keywords:** Urinary bladder dysfunction, Neurogenic bladder, Bladder augmentation, Ileocystoplasty, Biomaterials, Harnblasendysfunktion, Neurogene Harnblase, Blasenaugmentation, Ileozystoplastie, Biomaterialien

## Abstract

Bladder augmentation is a demanding surgical procedure and exclusively offered for selected children and has only a small spectrum of indications. Paediatric bladder voiding dysfunction occurs either on a basis of neurological dysfunction caused by congenital neural tube defects or on a basis of rare congenital anatomic malformations. Neurogenic bladder dysfunction often responds well to a combination of specific drugs and/or intermittent self-catheterization. However, selected patients with spinal dysraphism and children with congenital malformations like bladder exstrophy and resulting small bladder capacity might require bladder augmentation. Ileocystoplasty is the preferred method of bladder augmentation to date. Because of the substantial long-and short-term morbidity of augmentation cystoplasty, recent studies have tried to incorporate new techniques and technologies, such as the use of biomaterials to overcome or reduce the adverse effects. In this regard, homografts and allografts have been implemented in bladder augmentation with varying results, but recent studies have shown promising data in terms of proliferation of urothelium and muscle cells by using biological silk grafts.

## Zusammenfassung

Die Harnblasenaugmentation ist eine anspruchsvolle chirurgische Operation, die betroffenen Kindern unter bestimmten Voraussetzungen vorbehalten ist. Die kindliche Harnblasenentleerungsstörung basiert überwiegend auf angeborenen Neuralrohrdefekten und seltener auf angeborenen Defekten mit fehlentwickelter Harnblase. Die Dysfunktion bei neurogener Blase ist sowohl gut medikamentös als auch mit der sog. intermittierenden Selbstkatheterisierung behandelbar. Jedoch kann die Blasenaugmentation für ausgewählte Patienten mit spinaler Dysraphie und Kinder mit angeborenen Fehlbildungen wie Blasenekstrophie zur Vergrößerung der Blasenkapazität erforderlich sein. Die derzeitig am häufigsten verwendete Ileozystoplastie hat erhebliche unerwünschte postoperative Nebeneffekte. Zur Reduzierung dieser substanziellen Lang- und Kurzzeit-Komorbiditäten wird derzeit an neuen Techniken unter Verwendung von Homografts und Allografts geforscht, wobei aktuell auch zunehmend die Verwendung von Biomaterialien wie biologischen Transplantaten aus Seide untersucht wird, die eine Einsprossung von körpereigenem Urothel und Muskelzellen erlauben könnten.

## Introduction

Loss or malfunction of the lower urinary tract may cause urinary incontinence and chronic renal failure. The most common underlying conditions are spinal dysraphism (spina bifida), congenital malformations (exstrophy-epispadias complex, cloacal malformations) and trauma. Modern treatment of lower urinary tract dysfunctions consists of clean intermittent catheterization (as proposed by Lapides in 1972 [1]), medical treatment (anticholinergic medication and botulinum toxin A [2, 3]) and surgical reconstruction (augmentation cystoplasty, creation of a catheterizable conduit [4, 5]).

In this article we review various conditions and surgical options, and highlight new concepts for the use of biomaterials and tissue engineering in the field of urinary bladder reconstruction.

## Clinical presentation and issues

### Neuropathic bladder

Neural tube defects represent one of the most common birth defects (33–52/100,000 live births [6, 7]) as well as the most common cause of neurogenic bladder dysfunction [8]. In this regard, there is high accuracy and precision for obtaining the diagnosis by antenatal ultrasound [9].

Clinical presentation of neuropathic bladder includes incontinence, recurrent urinary tract infection and, if left untreated, chronic renal failure and end-stage renal disease [10]. Bladder dysfunction is caused by detrusor and/or sphincter over- and underactivity (detrusor sphincter dyssynergy). A high-pressure and low-compliance bladder causes destruction of the bladder architecture, leading to diverticulation and loss of contractility, subsequently to vesicoureteral reflux, chronic renal failure and incontinence [10, 11].

### Congenital malformations

Several rare anatomic malformations of the urogenital tract can cause bladder dysfunction as well, and are often diagnosed via prenatal ultrasound or magnetic resonance imaging (MRI) [12, 13]. Urogenital malformations that might require bladder augmentation include cloacal exstrophy (~0.19/100,000 live births [14, 15]) and bladder exstrophy (~3.3/100,000 live births [12]). In both entities, the volume of the urinary bladder is compromised, as is the compliance of the bladder wall. Again, insufficient treatment can lead to renal impairment [13].

### Therapeutic options

Modern treatment of lower urinary tract dysfunctions consists of

- Clean intermittent catheterization (CIC)
- Medical treatment (anticholinergic medication and botulinum toxin A)
- Surgical reconstruction

#### Clean intermittent catheterization

Clean intermittent self-catheterization (CIC) was introduced in 1972 and revolutionized the treatment of bladder dysfunction [1, 16]. CIC effectively lowers the intravesical pressure, provides urinary continence and consequently acts as protection against renal failure. It is the baseline treatment of bladder dysfunction and is also used in children with malformations of the exstrophy complex in addition to surgical management [17].

#### Pharmacological non-surgical treatment

Anticholinergic oral medication (i. e. oxybutynin) and muscle relaxation drugs in combination with intermittent self-catheterization poses an excellent option for long-term treatment in cases with neurogenic bladder dysfunction [18–20]. Side effects of the medical treatment include anticholinergic symptoms like drowsiness, flushes and palpitations. Additionally, a high compliance is needed, but in 75–90% [2, 20] of all patients with neurogenic bladder dysfunction, this non-surgical treatment shows good results. In case of persistent high intravesical pressure, submucosal injection of botulinum toxin A is implemented [21, 22].

#### Surgical treatment

##### Bladder augmentation

If medical treatment and/or interventional methods have failed, and high intravesical pressure and urinary incontinence or recurring urinary tract infections persist combined with present vesicoureteral reflux and impaired renal function, surgical treatment in terms of bladder augmentation is indicated [8, 23]. Urinary bladder augmentation-reconstruction includes and simplifies:

- Augmentation of the bladder capacity
  - via enterocystoplasty
  - or autoaugmentation
- treatment of incontinence
- catheterizable conduit (Mitrofanoff appendicovesicostomy)

The median age of children with neurogenic bladder dysfunction who undergo bladder augmentation is 12 years and more than half of these patients have spina bifida as the underlying disease [5]. In anatomical malformations, bladder augmentation is considered earlier than in children with neurogenic bladder dysfunction; the median age of those children is 6.4 years [15, 24]. The success rate of bladder augmentation with regard to the increase in bladder capacity as well as reduced intravesical pressure is high. In addition, the progression or the occurrence of kidney dysfunction can be avoided. Nonetheless, bladder augmentation requires close monitoring and further treatment of incontinence, and, thus, creation of a catheterizable Mitrofanoff conduit by use of the appendix vermiformis or small bowel is often part of the augmentation procedure [13]. In this regard, a catherizable urinary stoma may be crucial in the upkeep of the patient’s compliance to prevent short- and long-term complications such as mucous plugging and chronic renal disease [25].

**Fig. 1:**
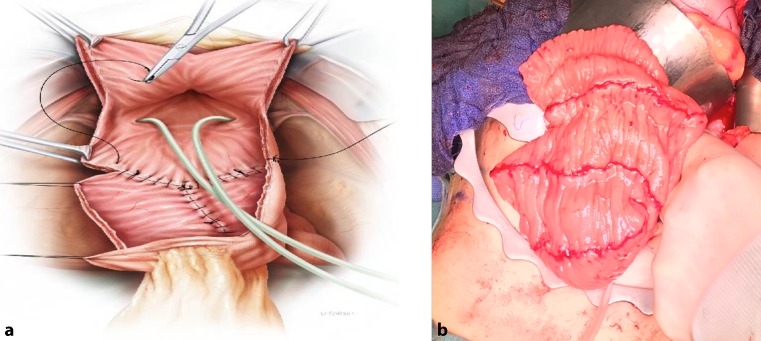
Scheme (**a**) and intraoperative view (**b**) of Ileocystoplasty. (Painting in **a** with Courtesy of Stephan Spitzer [http://www.spitzer-illustration.com/], Stephan Spitzer© all rights reserved)

Ileum is most commonly used to perform an augmentation cystoplasty (Fig. 1), followed by colon and stomach [5, 15]. Complications include small bowel obstruction, haematuria-dysuria syndrome, and an increase of urothelial cell carcinoma [5, 26]. In rare cases, the ureter can also be used for cystoplasty. However, ureteral dilatation with ipsilateral impaired renal function with no vesicoureteral reflux are prerequisites [5]. In even less cases, autoaugmentation (vesicomyectomy or vesicomyotomy) can be performed, but only in patients with a preoperative bladder volume of 75–80% compared to normal sized bladders of healthy individuals. Fibrosis and regrowth of the detrusor muscle with need for surgical revision have been described [5].

Although the incidence of short-and long-term complications is high, patients report a significant improvement in their quality of life [13].

##### Current risk factors and morbidity

Current risk factors and morbidity associated with bladder augmentation by non-urothelial tissue are shown in Table 1.

**Table 1:** Complication profile in bladder augmentation using gastrointestinal tissue

Complication	Incidence (%)
Bowel obstruction	3.2–10.3
Bladder calculi	15–40
Bladder perforation	2–8.6
Excessive mucous production	−100
Metabolic acidosis, metabolic deterioration	−100
Malignant transformation of bowel/tumour formation	0.5–10

Exposing non-urothelial tissue to urine causes a complex of problems. Through the constant contact of intestinal mucosa with urine, the accumulation of nitrosamines and infections can lead to premalignant and malignant degeneration in the long-term with incidences of 1.2–10.3% [5, 27–33]. In a recent review, Husmann et al. suggested that the primary bladder dysfunction as opposed to the augmentation cystoplasty is responsible for the development of intravesical neoplasia [25]. Moreover, bladder calculi with 15–40% [5, 34] as well as metabolic dysfunction with up to a 100% incidence rate can occur [34–36]. Enterocystoplasty (gastrocystoplasties and ileocystoplasties) increases the risk of small bowel obstruction with a rate up to a 3.2–10.3% [5, 37, 38]. Bladder perforation rate ranges from 2 to 8.6% [5]; additionally, spontaneous bladder rupture can occur in 3% and is connected to poor catherization compliance [25]. Excessive mucous production seems to occur more frequently after colocystoplasties and less frequently after ileocystoplasties and gastrocystoplasties [34, 39, 40].

Renal scarring and chronic renal failure in patients who undergo augmentation cystoplasty poses a long-term complication that seems to be linked to incompliance with self-catherization as well as catherization per urethra [25].

The implementation of an urothelium-like tissue might avoid such short-and long-term adverse effects (Table 1). Moreover, a high level of elasticity, a good compliance to high and rapid changes of the affecting pressure would be essential requirements to the used tissue. In addition, the potential development of a malignant degeneration of the used tissue should be excluded.

#### Animal testing in experimental bladder augmentation

##### Naturally derived biomaterials

- *Fascia and muscle grafts *(Table 2)
  Experimental bladder augmentation with muscle or muscle-backed flaps has been done as early as the beginning of the last century. In 1917, Neuhof performed bladder augmentation in dogs utilizing free fascia grafts [41]; details on the outcome are not available.
  Several experimental animal studies have since been performed on muscle-backed peritoneum, as well as rectus abdominis muscle flaps in rats [42]. Weingarten et al. found an increase in bladder volume in dogs after myoperitoneocystoplasty [43]. Manzoni et al. performed augmentation cystoplasty in thirty rats using rectus abdominis grafts, but bladder stones, chronic inflammatory response at sutures sites as well as undesirable residual muscle contractility occurred [42].
- *Demucosalized intestinal tissue *(Table 3)
  Since the 1980s, animal studies (rats, calves, rabbits and pigs) focussing on using demucosalized colonic tissue and small intestine have been performed. Weingarten et al. found increased bladder volumes as well as urothelial growth [43].
  Oesch et al.. [44] performed augmentation cystoplasty using stripped coecum and reported urothelial growth in only approximately half of the test rats after 4 months [44]. Motley et al. described urothelial growth in 10 out of 11 calves after sigmoidocystoplasty, but residual intestinal mucosa and graft diverticulation occurred [45]. Niku et al. showed incomplete urothelial growth in rabbits after colocystoplasty, leading to postoperative demise of several test animals due to inflammation [46]. Clementson Kockum et al. stated that de-epithelialized colocystoplasty leads to graft contraction, fibrosis and metaplasia in a study done on 21 piglets [47].
  Burgu et al. tried to overcome the side effects of ileocystoplasty by either adding gastric tissue or by performing reversed in situ ileocystoplasty in rats. There was no improvement in terms of metabolic imbalances and stone formation following gastroileocystoplasty and ileocystoplasty [48].
- *Other autologous tissue *(Table 4)
  Thangappan et al. successfully performed augmentation cystoplasty in 12 rats using de-epithelialized bladder wall grafts, although chronic inflammation as well as residual donor urothelial cells were found [49].
  Although augmentation with live-related-donor bladder grafts in rats showed promising results, transferring this particular two-step procedure to humans appeared difficult, as stated by Yamataka et al. [50].
  Ureterocystaugmentation was performed successfully in pigs, but only after iatrogenic creation of megaureters as reported by Ikeguchi et al. [51].
  Human dura mater, stomach and de-epithelialized small intestine tissue were used in augmentation cystoplasty in rabbits performed by Cranidis et al. The grafts covered iatrogenic bladder diverticuli following dertrusorectomy. The best results were obtained by using small intestine, although remnant intestinal mucosa was described. Graft contraction as well as bladder stones and fibrosis occurred in the groups with use of de-epithelialized gastric tissue and dura mater [52].
  To bypass risks and complications affiliated with gastric or intestinal resection, pedicled gastrocystoplasty was performed in a rodent model in 2004. Unfortunately, this method showed a number of side effects such as bladder calculi, metaplasia, passing of the test animal as well as scarring [53, 54].
  Dapena et al. demonstrated that hysterocystoplasty entails less adverse effects in animal studies than conventional enterocystoplasties in terms of metabolic imbalances and bladder calculi. However, smooth muscle cells were found to be sparse and there was evidence of fibrosis leading to obstruction [55–57].
  In a rat model, human amniotic membranes were successfully tested as hypoallergenic grafts, but the bladder capacity did not increase after application of amniotic membranes for augmentation because of the small size of the defect in the bladder wall and graft [58].

**Table 2:** Fascia and muscle grafts in experimental bladder augmentation

Year	Author	Animal	Graft material	Adverse effect
1917	Neuhof [41]	Dogs	Muscle fascia	–
1990	Weingarten et al. [43]	Ferrets	Myoperitoneal pedicle flap	Bladder stones
2001	Manzoni et al. [42]	Rats	Autoaugmentation and muscle flaps	Bladder stones, chronic inflammation

**Table 3:** Demucosalized intestinal tissue in experimental bladder augmentation

Year	Author	Animal	Graft material	Adverse effect
1988	Oesch et al. [44]	Rats	De-epithelialized coecum	Incomplete urothelial coverage
1990	Motley et al. [45]	Calves	Sigmoid	Graft diverticulation, residual intestinal mucosa
1995	Niku et al. [46]	Rabbits	Colon	Postoperative mortality, inflammation
1999	Clementson Kockum et al. [47]	Piglets	De-epithelialized colon	Graft contraction, fibrosis, metaplasia
2011	Burgu et al. [48]	Rats	Ileum, gastric tissue	Metabolic imbalances, bladder stones

**Table 4:** Various types of tissue used in experimental bladder augmentation

Year	Author	Animal	Graft material	Adverse effect
2012	Thangappan et al. [49]	Rats	De-epithelialized bladder wall grafts	Chronic inflammation, residual donor cells
2003	Yamataka et al. [50]	Rats	Bladder wall grafts	Two-step procedure and immunosuppressants may be required
1998	Ikeguchi et al. [51]	Pigs	Ureteral tissue	Megaureter required
1998	Cranidis et al. [52]	Rabbits	Human dura mater, de-epithelialized small intestine and gastric tissue	Residual intestinal mucosa, stomach perforation, graft contraction
2004	Aslan et al. [53]	Rat	Pedicled gastric tissue	Bladder stones, metaplasia, postoperative mortality, scarring
2012, 2013	Dapena et al. [55, 56]	Rat	Uterus	Fibrosis
2017	Barski et al. [58]	Rat	Human amniotic membrane	No increase in capacity

##### Cell-seeded biological grafts

Studies investigating cell-seeded biological grafts are outlined in Table 5.

**Table 5:** Seeded de-epithelialized intestinal tissue in experimental bladder augmentation

Year	Author	Animal	Graft material	Adverse effect
2001	Blanco Bruned et al. [59]	Rats	Seeded intestinal grafts	No increase in capacity
2005	Hafez et al. [60]	Pigs	Seeded demucosalized colon	No information on results with neuropathic bladder cells
2015	Hidas et al. [61]	Pigs	Seeded demucosalized colon	No information on results with neuropathic bladder cells
2004	Fraser et al. [62]	Minipigs	Seeded de-epithelialized uterine tissue/colon	Incomplete urothelial coverage, graft contraction, fibrosis
2011	Turner et al. [63]	Pigs	De-epithelialized colon, urothelium sheets	Graft shrinkage

Schaefer et al. succeeded in transferring urothelial cells to colon and gastric grafts in vitro. However, they did not perform augmentation cystoplasty in vivo [64].

No increase in postoperative bladder volume was found following intestinal grafts seeded with urothelium in rats as demonstrated by Blanco Bruned et al., which resulted in a high mortality rate of 63.3% [59].

Seeding colonic grafts with urothelial cells and smooth muscle cells with an aerosol spraying technique was developed in 2003 [65]. Two ensuing studies showed the effectiveness of this bladder augmentation method in terms of cell adhesion and confluent epithelial coverage, although smooth muscle cell growth occurred only after an additional adding of detrusor cells to the urothelial cells [60, 61]. Hafez et al. compared aerosol transfer of smooth muscle cells onto demucosalized colon grafts to conventional colocystoplasty and found complete urothelial coverage only in the animals that underwent the aerosol graft cystoplasty [60]. Hidas et al. showed similar results, with no fibrosis or inflammation in porcine cystoplasty using the aerosol transfer technique. However, Hidas et al. stated that further studies in animals with neuropathic bladder should be done, as the results in a neuropathic bladder population might deviate [61].

Incomplete urothelial covering of seeded de-epithelialized uterine tissue derived from minipigs was reported in an experimental cystoplasty study done by Fraser et al., as well as graft contraction and fibrosis in de-epithelialized colonic tissue. [62].

Turner et al. successfully combined urothelium sheets with de-epithelialized colon tissue in a porcine model, but described graft shrinkage [63].

- *Acellular matrix grafts *(Table 6)
  Biomaterials such as acellular matrix grafts and bladder submucosa collagen matrix have been used successfully in animal trials because of their good biocompatibility [66]. However, acellular bladder matrix grafts do not promote the ingrowth of smooth muscle cells and there is therefore no structural integration. Postoperative urinoma and urinary tract infection can occur as well, and antigenicity cannot be precluded completely [67–70].
  Kropp et al. described complete urothelial graft overgrowth in 22 rats after cystoplasty with porcine-derived small intestinal submucosa (SIS); however, there were bladder calculi, leakage, inflammation and incomplete smooth muscle cell growth noticed [71].
  Parshotam Kumar et al. reported on the evidence of fibrosis with SIS in augmentation cystoplasty in lambs [72].
  Sharma et al. seeded stem cells unto de-epithelialized small intestine tissue and used the graft to perform augmentation cystoplasty in primates, showing urothelial and smooth muscle growth but no increase in postoperative bladder volume [73].
  By managing to perform a bladder augmentation using only smooth-muscle cells sheets, Talab et al. showed that neovascularization and epithelialization can be achieved without the use of a scaffold. However, there was no testing regarding the postoperative bladder volume. [74].
  Muscle cell migration could also be enhanced by seeding acellular bladder grafts with adipose-derived stem cells, as demonstrated by Zhe et al., although bladder calculi and insufficient smooth muscle cell growth occurred [75].
  Smooth muscle cells harvested from neuropathic bladders showed similar results to matrices seeded with normal cells when seeded unto matrices in vitro and then transplanted in vivo [76, 77]. This adds a new perspective to the findings of Subramaniam et al., who found that urothelial cells harvested from patients with bladder dysfunction showed reduced proliferation and differentiation [78].
- *Polymers, collagen grafts, glycosaminoglycans *(Table 7)
  Nano-structured polymers have been tested since 2007, providing adequate surface properties for smooth muscle and urothelium proliferation. However, death as a consequence of bladder leak was reported as well as untimely biodegradation, sparse smooth muscle cell growth, fibrosis as well as no increase of the bladder capacity [79, 80]. However, seeded polymer grafts did not show these adverse effects as shown by Kwon et al. [80]. Parshotam et al. found that augmentation cystoplasty using INTEGRA® (INTEGRA LIFE SCIENCE CORPORATION, Plainsboro, New Jersey, USA) collagen matrix showed better results than SURGISIS® (COOK, Spencer, Indiana, USA) collagen matrix or demucosalized enterocystoplasty in lambs. Mucous cysts as well as intestinal obstruction, fibrosis and graft shrinkage were described in the enterocystplasty as well as in the SURGISIS® (COOK, Spencer, Indiana, USA) groups [72].
  Zhou et al. advanced the use of tissue-engineered grafts by applying vascular endothelial growth factor and platelet-derived growth factor onto bladder acellular matrices to enhance muscle and vascular ingrowth. While the bioactive factors did promote smooth muscle cell regeneration and neovascularization, urinary leakage and bladder stone formation occurred, as well as graft shrinkage, scarring and graft calcification [81].
  Further experimentation by Vardar et al. on collagen–fibrin scaffolds showed improved urothelialization and smooth muscle cell growth by adding insulin-like growth factor. Nonetheless, there was hypertrophy of the constructed urothelium which could lead to outlet obstruction [82].
  In a recent study from 2017, unseeded and seeded cystoplasty collagen grafts were compared, with inconsistent results regarding the ingrowth of urothelial and smooth muscle cells [83].

**Table 6:** Acellular matrix grafts in experimental bladder augmentation

Year	Author	Animal	Graft material	Adverse effect
1995	Kropp et al. [71]	Rats	SIS	Bladder stones, inflammation, leakage, inflammation, incomplete smooths muscle cell growth
2010	Parshotam Kumar et al. [72]	Sheep	SIS	Fibrosis
2011	Sharma et al. [73]	Primates	Seeded SIS with stem cells	No increase in capacity
2014	Talab et al. [74]	Rabbits	Smooth-muscle cell sheets	Fibrosis, no information on postoperative bladder capacity
2016	Zhe et al. [75]	Rats	ACS-seeded acellular bladder grafts	Bladder stones, insufficient cell growth

*SIS* small intestinal submucosa, *ACS* adipose-derived stem cells

**Table 7:** Polymers, collagen grafts, glycosaminoglycans in experimental bladder augmentation

Year	Author	Animal	Material	Adverse effect
2007	Pattison et al. [79]	Rats	Polymer scaffolds	Bladder leak, bladder stones postoperative mortality, slow biodegradation, mechanical difficulties
2008	Kwon et al. [80]	Dogs	Polymer scaffold	Chronic inflammation, rapid scaffold degradation
2010	Parshotam Kumar et al. [72]	Lambs	Collagen scaffolds (INTEGRA® [INTEGRA LIFE SCIENCE CORPORATION, Plainsboro, New Jersey, USA], SURGISIS® [COOK, Spencer, Indiana, USA])	Fibrosis, graft contraction
2013	Zhou et al. [81]	Rabbits	Acellular bladder matric and growth factors	Bladder stones, graft shrinkage/calcification/scarring, urinary leakage
2016	Vardar et al. [82]	–	Collagen–fibrin scaffold and IGF-1	Possible outlet obstruction because of tissue hypertrophy
2017	Leonhäuser et al. [83]	Minipigs	Unseeded and seeded collagen scaffolds	Inconsistent cell ingrowth, risk of leakage

##### Synthetic materials

Synthetic materials which have been employed in experimental bladder augmentation are presented in Table 8.

**Table 8:** Synthetic materials in experimental bladder augmentation

Year	Author	Animal	Material	Adverse effect
1957	Kudish [84]	Dogs	Polyvinyl sponges	Foreign body reaction
1970	Kelâmi et al. [85]	Dogs	Teflon® (BARD INC., Murray Hill, New Jersey, USA) felt	Fibrosis, incomplete urothelial coverage, no smooth muscle cell growth, graft collapse
1994	Virseda Chamorro et al. [86]	Dogs	Gore-Tex® (W.L. GORE and ASSOCIATES, INC., Flagstaff, Arizona, USA)	No increase in capacity

Polyvinyl sponges [84], Teflon® (BARD INC., Murray Hill, New Jersey, USA; [85]), as well as Gore-Tex® (W.L. GORE and ASSOCIATES, INC., Flagstaff, Arizona, USA); patches [86] have been used in experimental animal studies, too. However, there was no increase in capacity due to the stiffness of the material and no ingrowth of muscle cells [86]. Moreover, foreign body reactions, fibrosis, incomplete urothelial growth as well as no smooth muscle cell ingrowth have been described [84, 85].

##### Silk-based scaffolds

Silk-based scaffolds which have been used in experimental bladder augmentation are shown in Table 9.

**Table 9:** Silk scaffolds in experimental bladder augmentation

Year	Author	Animal	Material	Adverse effect
2013	Seth et al. [87]	Rat	Silkworm silk scaffold/combined with SIS	Foreign body reaction
2014	Chung et al. [88]	Rat	Silkworm silk fibroin scaffolds combined with SIS	Bladder stones, bladder rupture, chronic inflammation, residual silk
2015	Zhao et al. [66]	Rat	Silkworm silk combined with acellular bladder matrix graft	No increase in capacity
2013	Tu et al. [89]	Pigs	Acellular silkworm silk scaffolds	Urinary leakage, bladder calculi, graft contraction

*SIS* small intestinal submucosa

To facilitate the growth of urothelial and smooth muscle cells, grafts have been lined with silkworm silk (*Bombyx mori*). However, Seth et al. described a high incidence of foreign body reaction in either silk matrices or small intestinal submucosa combined with silkworm silk [87]. Chung et al. found regrowth of smooth muscle cells by combining small intestinal submucosa and silkworm silk in a rat cystoplasty model, but again, bladder stones and bladder rupture as well as chronic inflammation and residual silk were present [88]. Zhao et al. used silkworm silk to line a bladder acellular matrix graft in rats. Although there was ingrowth of smooth muscle cells present, bladder stones as well as graft perforation and chronic inflammatory response occurred [66]. Tu et al. tested acellular silkworm silk scaffolds in pigs, and although smooth muscle cells as well as nerve cells and neovascularisation were successful, urinary leakage as well as urinary calculi and graft contraction still ensued [89].

Due to the fact of current inflammation, stone production, leakage and persistence of silk graft remnants, this particular method for bladder augmentation needs to be further investigated. Presumably, a more suitable silk donor has to be found to eliminate these adverse effects such as antigenicity and formation of calculi.

## Conclusion and perspective

In conclusion, the implementation of tissue and neo-organs fabricated in vitro seems feasible in partial or total organ reconstruction. Seeded biomaterials (collagen, keratin, alginate, acellular tissue matrices, synthetic polymers) surpass non-biomaterials (Teflon® [BARD INC., Murray Hill, New Jersey, USA], silicone, Vicryl® [ETHICON INC., Cincinetti, Ohio, USA], polyvinyl, unseeded collagen matrices) in terms of biocompatibility, degradation, cell adhesion substrate, tissue development, mechanical and physical properties as well as plasticity.

The best results in seeding the grafts are obtained by using autologous urothelial and smooth muscle cells.

Research concerning the use of stem cells, amniotic fluid and progenitor cells from urine as well as “printing” 3D scaffolds in vitro is still ongoing [76, 77, 90].

Minimizing or alleviating the ailments of congenital malformations or traumatic injuries of the urinary tract by operative means remains a challenge for paediatric surgeons as well as for interdisciplinary carers.

New techniques and possibilities in the operating field of bladder augmentation and ureteral reconstruction give new future perspectives in terms of reducing side effects and maximizing the quality of life of the afflicted patients.

## Abbreviations

CIC: Clean intermittent self-catheterization
MRI: Magnetic resonance imaging
SIS: Small intestinal submucosa
